# Physical Activity and Obesity in Canadian Women

**DOI:** 10.1186/1472-6874-4-S1-S6

**Published:** 2004-08-25

**Authors:** Shirley Bryan, Peter Walsh

**Affiliations:** 1 Centre for Chronic Disease Prevention and Control, Health Canada, 120 Colonnade Rd, Ottawa, Canada, K1A 0K9; 2 Centre for Chronic Disease Prevention and Control, Health Canada, 120 Colonnade Rd, Ottawa, Canada, K1A 0K9

## Abstract

**Health Issue:**

Overweight and  obesity have been recognized as major public health concern in Canada and throughout the world. Lack of physical activity, through its impact on energy balance, has been identified as an important modifiable risk factor for obesity. Physical activity and obesity are also important risk factors for a variety of chronic diseases. This chapter provides an overview of the current state of physical activity and overweight/obesity among Canadian women.

**Key Findings:**

For all ages combined more women (57%) than men (50%) are physically inactive (energy expenditure <1.5 KKD). Physical activity increases as income adequacy and educational level decrease. Physical inactivity also varies by ethnicity. The prevalence of both overweight (BMI 25.0 – 29.9 kg/m^2^) and obese (BMI ≥ 30 kg/m^2^) Canadian women has increased 7% since 1985. Obesity increases with age and is highest among women reporting low and lower middle incomes and lower levels of education. The prevalence of obesity is highest among Aboriginal women and men (28% and 22% respectively).

**Data Gaps and Recommendations:**

There is currently no surveillance system in Canada to monitor the level of physical activity among children, those performing activity at work, at school or in the home. There is a gap in the knowledge surrounding socio-cultural and ecological determinants of physical activity and obesity and the associations of these to chronic disease among women and minority populations. Multi-sectoral policy interventions that act to decrease the broad systemic barriers to physical activity and healthy weights among all women are needed.

## Background

Overweight and obesity have been recognized as a major public health concern not only in Canada but also throughout the world. Physical activity, through its impact on energy balance, has been identified as an important modifiable risk factor for obesity. Both lack of physical activity and obesity are also important independent risk factors for the development of many chronic diseases that affect women and thereby place a substantial burden on the health care system. Despite this knowledge, the prevalence of obesity continues to increase among women, and only a small proportion of the female population is active enough to achieve health benefits.

The aim of this chapter is to provide an overview of the current state of physical activity and overweight/obesity among Canadian women. The health benefits of regular physical activity are also briefly reviewed. Attention is paid to the individual and systemic determinants of women's adoption of regular physical activity throughout their lifespan. A summary of the current Canadian recommendations for physical activity and the World Health Organization (WHO) recommendations for obesity prevention through regular physical activity are also provided. A detailed interpretation of the Canadian Community Health Survey provides prevalence rates of physical inactivity, overweight and obesity, and these are analyzed in relation to gender, socio-economic status, educational level and race. An analysis of trends is presented where the data are available.

## Increasing Trends in Overweight and Obesity

The WHO reports that overweight and obesity are increasing worldwide at an alarming rate, in both developed and developing countries, and this trend is seen among adults and children alike. [1] Although genetic susceptibility may explain up to 40% of the obesity phenotype, [2] technological, lifestyle and cultural changes over the past 50 years are being implicated as the most likely cause of the recent obesity epidemic. [1] The most important factors associated with increased risk of overweight and obesity are physical inactivity and high-energy, dense diets over the medium or long term. It is becoming increasingly clear that maintenance of a healthy adult weight, through proper balance of caloric intake and regular physical activity, is a key factor in chronic disease prevention.

## Economic Costs of Inactivity and Obesity

Physical inactivity and obesity, through their association with the development of chronic diseases, place a significant economic burden on the health care system. In 1999, $2.1 billion in direct health care costs in Canada were directly attributable to physical inactivity, equalling 2.5% of the total health care expenditures for that year. [3] In a study by Birmingham et al. [4] the total direct cost of obesity in Canada in 1997 was estimated to be over $1.8 billion, or 2.4% of the total health care expenditures for all diseases that year. Katzmarzyk et al. [3] inflated this number to reflect 1999 dollars and found that obesity accounted for approximately $2.0 billion in direct health care expenditures, which is comparable to the estimates for physical inactivity for that year.

## Health Implications

Physical activity throughout the lifespan may independently enhance women's and girls' health through the reduction of chronic disease risk and improved quality of life. Specifically, being physically active may decrease a woman's risk of developing coronary artery disease, [5] type 2 diabetes, [6] hypertension, [7] osteoporosis [8] and colon/colorectal cancer. [9] Epidemiologic studies have shown that participation in regular physical activity may also be protective against cognitive impairment, [10] dementia, [11,12] breast cancer, [13] hip fractures, [14] and a range of psychological outcomes, including anxiety, depression, stress and low self-esteem. [15] Regular physical activity may also increase longevity [16] and reduce the risk of prolonged disability in old age. [17]

Although some decrease in function, strength and stamina are unavoidable with age, the rate of decline can be diminished through supportive environments that promote better health through healthy levels of physical activity. [18] Healthy physical activity levels among older women can act to decrease the amount of weight gain experienced during menopause and may attenuate the accumulation of adipose tissue in the upper and central body regions after menopause. [19] Physical activity in the form of vigorous exercise and resistance training of moderate intensity can also decrease the risk of osteoporotic fracture among women, by preserving bone density or modestly increasing bone mineral content at clinically relevant sites. [8,20] Furthermore, regular physical activity can reduce the risk of falls and hip fractures among older adults. [21]

Maintenance of a healthy weight in adulthood may also decrease a woman's risk of many adverse health outcomes. Overweight and obesity are associated with an increased risk of type 2 diabetes, [6] coronary heart disease, [22,23] hypertension, [24] some cancers [25-27] and premature mortality. [28] Epidemiologic studies have also noted a decreased risk of ischemic stroke, [29] sleep apnoea and hypoventilation syndrome, [30,31] gallbladder disease [32] and osteoarthritis [33] among adults who have undergone weight loss.

The health consequences of childhood obesity are less well understood; however, an association between childhood physical activity levels, obesity and early chronic disease indicators (e.g. physical fitness and high blood pressure) has been identified. [34] Appropriate amounts of physical activity during childhood and adolescence are critical for the development of peak bone mass, an important predictor of osteoporosis and osteoporotic fractures later in life. [35-37] Children who exercise habitually have stronger bones than their non-active peers, and exercising during puberty is particularly efficacious in producing strong bones. [38]

The prevalence of childhood obesity has increased rapidly in the Canadian population, and unhealthy behaviour patterns (sedentary lifestyle, unhealthy eating) are being blamed for this increase. [39] As a consequence, the incidence of obesity-related chronic disease (e.g. type 2 diabetes) and precursors to chronic disease (e.g. hypertension) are also increasing among children with high BMI values. [40] Additionally, childhood overweight/obesity may lead to undesirable body image and poor self-esteem. [41,42] The state of childhood overweight and obesity is a major public health concern, since, once developed, the unhealthy lifestyle habits adopted during childhood and adolescence not only result in early manifestations of chronic disease but also tend to follow that individual throughout the lifespan. [43]

Despite this knowledge and the increased interest in and participation by women in sport and recreational activity over time, only a small proportion of the female population is active enough to achieve health benefits. At all ages, women and girls are less likely than males to participate in physical activity, and the more intense the activity, the greater this disparity. [18] From a long-term health perspective, decreased rates of physical activity among adolescent girls and older women are particularly worrisome.

Involvement in physical activity is closely intertwined with the social, economic and health status of women, and is associated with unique individual and environmental determinants of physical activity that vary across life situations. The most commonly reported barrier to women's participation in physical activity is lack of time due to family responsibilities, including child care, cooking, cleaning and other household-related tasks. [44-46] Other barriers often reported include lack of motivation, poor perception of health, lack of self-efficacy, lack of support from employers and lack of social support. [45,47] Cultural barriers to participation among women of ethnic minorities may include lack of knowledge of the importance of exercise, [48] differing value ascribed to physical activity, [49] health problems, [50] acceptance of larger body sizes, [51] lack of community support, differing social norms, [47] language barriers and culturally dictated familial roles. [47] Living environment and socio-economic status are also associated with important barriers to physical activity for women. These may include lack of safe places to exercise, tiredness due to physically demanding jobs, limited access to affordable or adequate facilities, lack of social support and child care resources, and a lack of both community resources for equipment and gender-sensitive programs. [52]

It is not well understood why the rates of physical activity among adolescent girls are lower than among boys. It has been suggested that these rates may reflect girls' attitudes towards exercise, which are rooted in unrealistic stereotypes of the active female body and constant reminders of their physical limitations. [18] Many teenaged girls report a desire to change their physical appearance to that of the currently fashionable and heavily marketed fashion model ideal. [53] Consequently, some girls strive to use physical activity as a means to repair and reshape their bodies to fit this ideal. [18] However, this vision of an unattainable goal may also result in decreased activity levels in other girls. Among females participating in high-performance athletics, body weight can be seen as an important predictor of success, and disordered eating behaviours and exercise patterns can become problematic. [54] Decreased physical activity among adolescent girls may also reflect limited access to high school physical education classes that provide pleasure and skill development in a non-threatening atmosphere. [18] Physical education programs continue to focus on male-oriented Western team sports that emphasize strength, power, competition and contact. [55] Such a curriculum denies active role models to Canadian girls of varying cultural backgrounds. [18,56]

Lower rates of participation in physical activity among older women may reflect barriers related to knowledge of the physical activity and its benefits, fear of falling, lack of access, lack of social support and environmental factors (e.g. poor weather, safe places to exercise). Older women may also cite their declining health and their perception of being too old as reasons for not participating. [57]

To date, health promotion activities to address physical activity and obesity have not taken into account the systemic and cultural pressures that shape female participation in physical activity and the maintenance of a healthy body weight. [18] Current Canadian guidelines for physical activity, promoted through Canada's Physical Activity Guide to Healthy Active Living, have been developed to provide Canadians with the information they need at an individual level. [58] These recommendations state that adults should perform a minimum of 30 to 60 minutes of moderate activity (e.g. brisk walking, biking, swimming) on most days of the week, starting slowly and building up the duration over time. Evidence suggests that this level of activity, which can be realized through a variety of activities that fit into each individual's lifestyle, is sufficient for cardiovascular/metabolic health. [43] Increasing the duration and intensity of physical activity over time may have a greater effect on some health outcomes but may be viewed as an unattainable goal for some of the population. [43]

The WHO has recently released recommendations for individuals about the level of activity recommended for the maintenance of a healthy body weight through regular physical activity. These recommendations state that a total of one hour of moderate-intensity activity, such as walking, on most days of the week, particularly for people with sedentary occupations, will decrease the likelihood of becoming overweight. [43] Recommendations such as the Canadian guidelines and the WHO recommendations are an important part of reducing the barriers of physical activity at the individual level, but if real progress is to be made, public health programs must now address the systemic barriers to participation in physical activity and the maintenance of healthy weights that women face.

## Methods

Physical activity data were taken from the Canadian Community Health Survey (CCHS) 2000–2001 using the physical activity index variable. This is derived from an energy expenditure variable that is based on a number of questions dealing with self-reported leisure-time physical activity. The proportion of individuals classified as physically inactive was broken down by five-year age groups and sex. For the purpose of this report, physically inactive is defined as self-reported daily energy expenditure of less than 1.5 kilocalories per kilogram per day (KKD) and is equivalent to the energy expenditure associated with about one half-hour of walking at a leisurely pace. Additional analysis of those classified as physically inactive was performed according to income adequacy, highest level of education achieved and self-ascribed ethnicity.

**Figure 1:**
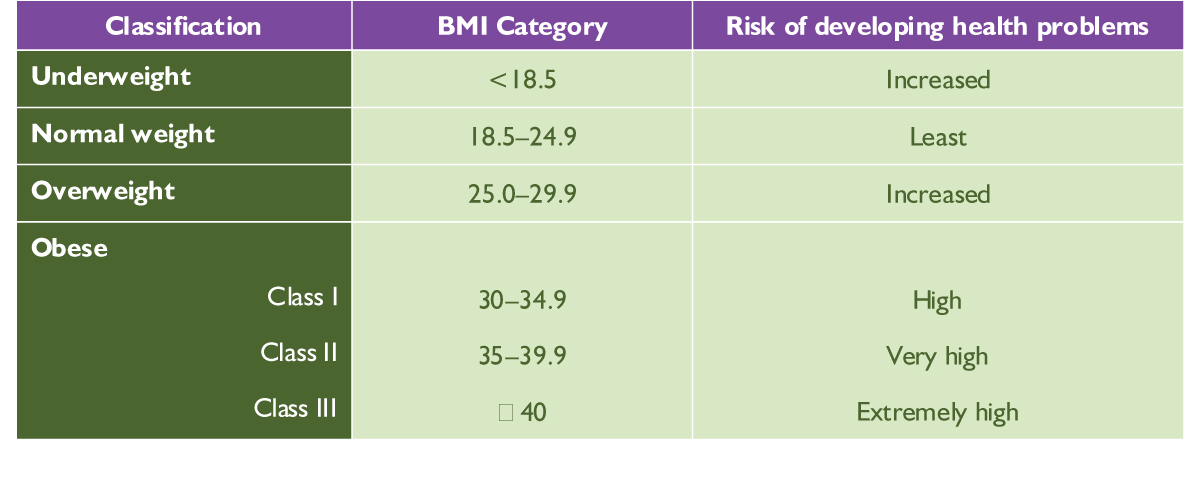
**Health Risk Classification according to Body Mass Index (BMI) for Use with Adults aged 18 and older. Not for use with pregnant or lactating women. **Source: Health Canada: Canadian guidelines for body weight classification in adults.

Overweight and obesity status is most commonly reported in relation to body mass index (BMI) values. BMI is a simple ratio of weight (measured in kilograms) to height (measured in metres), such that BMI (kg/m^2^) = wt/ht^2^. BMI guidelines for healthy weights were adopted in Canada in 1988 and have recently been updated to reflect the current WHO guidelines. As outlined in Figure 1, and for the purposes of this report, overweight is defined as a BMI value of 25.0–29.9 kg/m^2^ and obese is defined as a BMI of ≥ 30 kg/m^2^.

BMI was calculated from all non-pregnant respondents' self-reported weight and height. Individuals with a BMI value from 25.0 to 29.9 kg/m^2^ and a BMI value of 30 kg/m^2^ or more were classified for analysis by five-year age group and sex. Additional analysis of those classified as obese (BMI ≥ 30 kg/m^2^) was performed according to income adequacy, highest level of education achieved and self-ascribed ethnicity. BMI time trends are presented using data from the four national population surveys that collected self-reported height and weight and occurred at roughly five-year intervals: the 1985 and 1991 General Social Surveys (Health), the 1994–1995 National Population Health Survey and the Canadian Community Health Survey 2000–2001.

## Results

**Figure 2:**
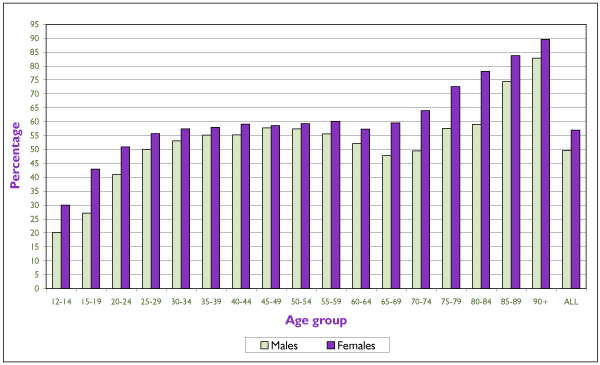
Percentage Physically Inactive* by Age Group and Sex, Canada, 2000 Source: Canadian Community Health Survey, 2000–2001. Statistics Canada

Data from the 2000–2001 CCHS indicate that 57% of females and 50% of males aged 12 years and older reported being physically inactive (Figure 2). This proportion has decreased slightly over time, down from 62% and 54% in 1994–1995 for women and men respectively (NPHS 1994–1995). For every age group, more females than males are inactive, and this sex disparity is greatest in the youngest and oldest age groups. These data indicate that 43% of girls who are of high school age (15 to 19 years) are inactive, a 13% higher proportion than that of girls aged 12 to 14 years. Although this relation is also seen for boys, the magnitude of change is much less (7%).

**Figure 3:**
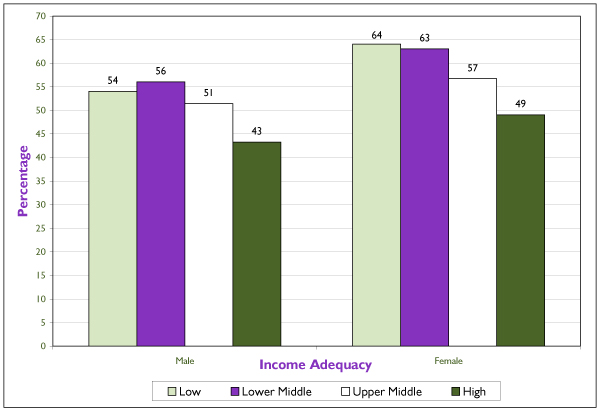
Percentage Physically Inactive by Sex and Income Adequacy* Canada, 2000 Source: Canadian Community Health Survey, 2000–2001. Statistics Canada

**Figure 4:**
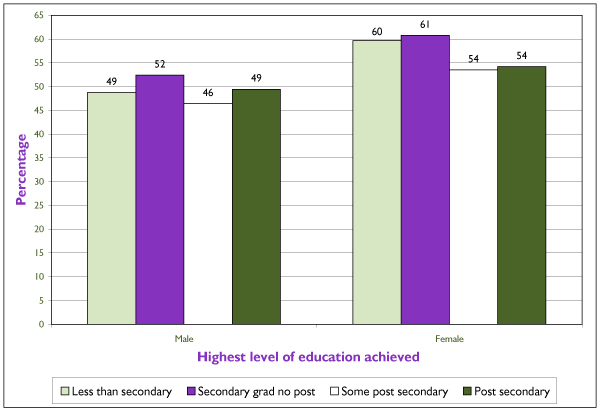
**Percentage Physically Inactive by Sex and Highest Level of Education, Canada, 2000 **Source: Canadian Community Health Survey, 2000–2001. Statistics Canada

**Figure 5:**
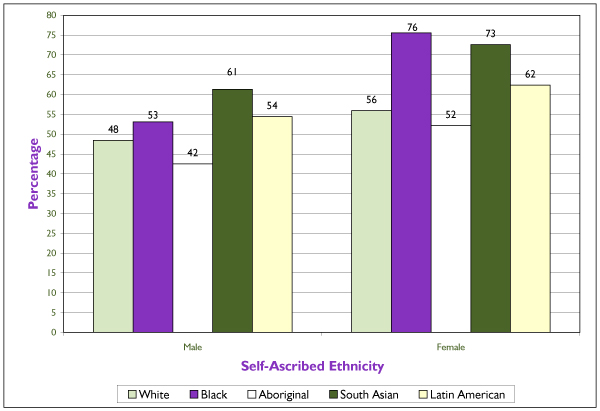
**Percentage Physically Inactive by Sex and Race, Canada, 2000 **Source: Canadian Community Health Survey, 2000–2001. Statistics Canada.

As women age they become progressively more inactive, and reported inactivity is alarmingly high in the older age groups: 72% of Canadian women over the age of 70 are considered inactive, as compared with 56% of men in the same age group. As depicted in Figures 3 and 4, physical inactivity increases as income adequacy and educational level decreases, and this relation is stronger for women than men. Physical inactivity varies with ethnic group; 76% of Black women and 73% of South Asian women were classified as inactive (Figure 5).

**Figure 6:**
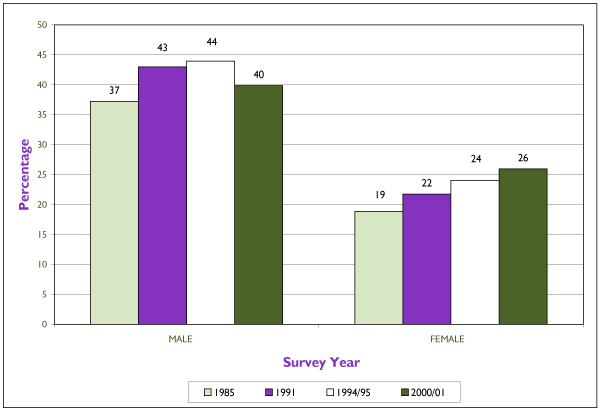
**Proportion of Canadians Aged 20–64 With BMI >= 25 and <30 by Sex and Year of Survey **Source: General Social Survey 1985 and 1991, National Population Health Survey 1994–1995, Canadian Community Health Survey, 2000–2001. Statistics Canada.

**Figure 7:**
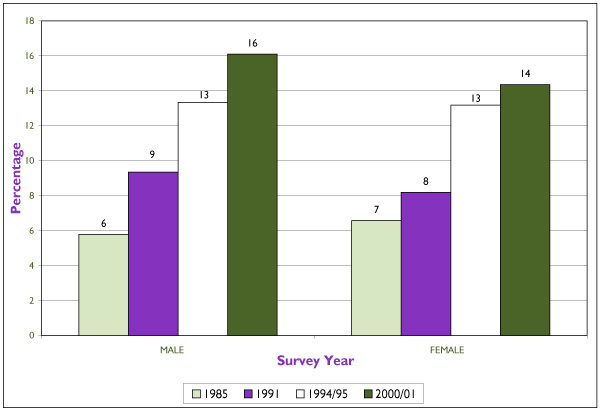
**Proportion of Canadians Aged 20–64 With BMI >= 30 by Sex and Year of Survey. **Source: General Social Survey 1985 and 1991, National Population Health Survey 1994–1995, Canadian Community Health Survey, 2000–2001. Statistics Canada.

**Figure 8:**
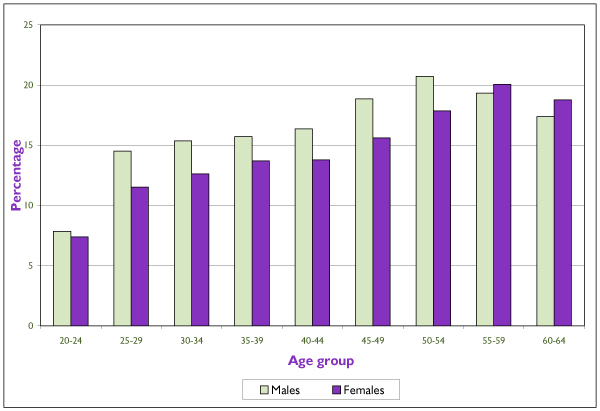
**Proportion of Canadians With BMI >= 30 by Sex and Five Year Age Group **Source: Canadian Community Health Survey, 2000–2001. Statistics Canada.

As depicted in Figure 6, the prevalence of overweight increased in women between 1985 and 2000–2001, from 19% to 26%. Although overweight among men has also increased since 1985, there has been a slight decrease in the prevalence over the last five years (down from 44% in 1994–1995 to 40% in 2000–2001). Canadian trend data indicate that the prevalence of obesity (BMI ≥ 30 kg/m^2^) has steadily increased, from 7% to 14% in women and from 6% to 16% in men, since 1985 (Figure 7). Analysis of five-year age groups reveals that the prevalence of obesity among women increases with age, peaking in the 55 to 59 age category and decreasing steadily thereafter (Figure 8). An increase in obesity with age also occurs in the male population, with a peak in the 50 to 54 age group. These data also reveal a substantial increase in the prevalence of obesity during the young adult years in both men and women. For both sexes, between the age categories 15 to 19 and 25 to 29 years the prevalence increased by about 8%, from 3.3% to 11.5% and from 6% to 14% in women and men respectively.

**Figure 9:**
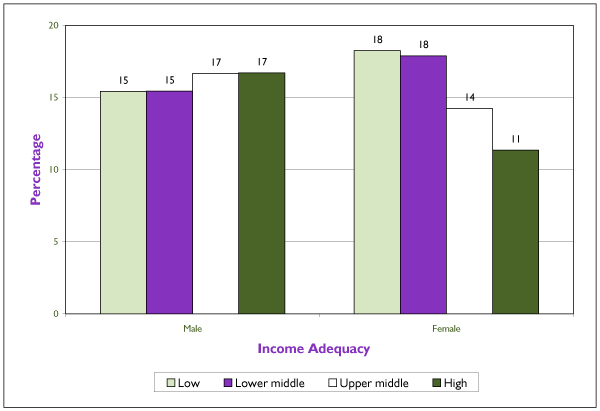
Percentage Obese (BMI >= 30) by Sex and Income Adequacy*Canada, 2000 Source: Canadian Community Health Survey, 2000–2001. Statistics Canada.

**Figure 10:**
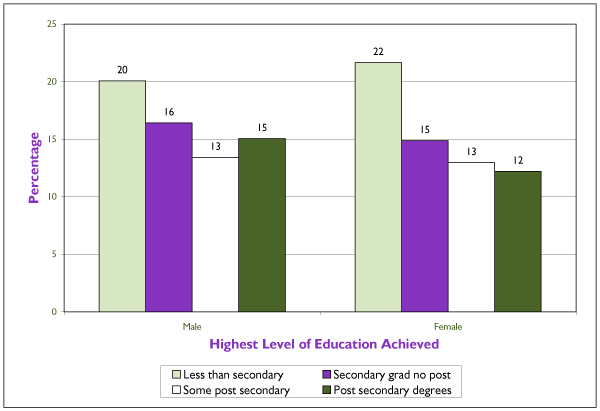
**Percentage Obese (BMI >= 30) by Sex and Highest Level of Education, Canada, 2000 **Source: Canadian Community Health Survey, 2000–2001. Statistics Canada.

**Figure 11:**
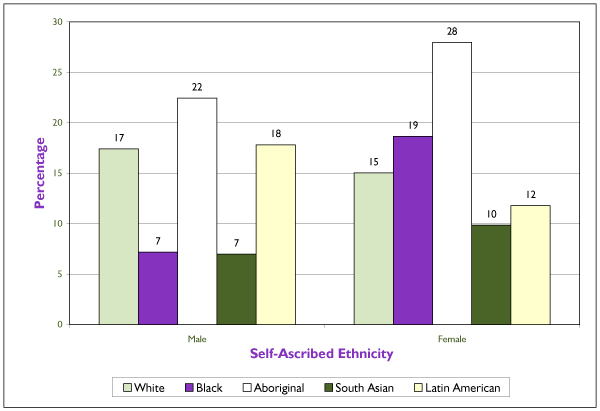
**Percentage Obese (BMI >= 30) by Sex and Race, Canada, 2000 **Source: Canadian Community Health Survey, 2000/01. Statistics Canada.

The prevalence of obesity is highest among women reporting low and lower middle incomes and women reporting lower levels of education (Figures 9 and 10). The reverse trend is seen among men: those with higher income and higher levels of education have the highest prevalence of obesity. The prevalence of obesity is high among Aboriginal women and men (Figure 11) as well as among Black women.

## Discussion

### Data Limitations

Data collection for the CCHS 2000–2001 occurred through random selection of one respondent per household who was 12 years of age or older, among a representative sample of Canadian households in all provinces, excluding the territories, Indian Reserves, Canadian Forces Bases and some remote areas. Therefore, these data are not representative of those populations in Canada that were not included in the survey. The CCHS is a telephone survey and is therefore limited to collection of self-reported data.

The physical activity index was derived from total energy expenditure, based on all leisure-time physical activities reported by the respondent. Physical activities performed at work (e.g. work-related labour activity), at school (e.g. physical education classes) or in the home (e.g. household chores) are not included in the calculation of this index, which may lead to an underestimate of the actual amount of activity performed by some respondents. In general, most studies show that self-reports do not provide accurate estimates of the absolute amount of physical activity. [59] Recalling past physical activity is a highly complex cognitive task, which may differentially affect the young and very old. [60] The use of ambiguous terms such as "physical activity," "moderate intensity" and "leisure time" may also affect a person's response, since the definition of these terms may differ between respondents and investigators. [59] Another limitation of self-reported physical activity levels is social desirability bias, which can lead to over-reporting of physical activity. [61] Finally, survey questions may limit or not assess the primary modes of activity for certain gender, age, cultural, occupational or income groups. Few self-reported measures have been developed for or validated in distinct demographic, ethnic or cultural groups. [59]

In the case of BMI, when individuals self-report these values they tend to overestimate their height and underestimate their weight, which leads to an underestimate of actual BMI value. [62] This under-reporting appears to be consistent across socio-economic strata but occurs more often among women and girls, [63] the elderly [64], and heavier and taller BMI respondents. [65] Conversely, underweight individuals tend to over-report weight. [65] Studies have concluded that self-reported data tend to underestimate the prevalence of overweight and obesity in a population by approximately 10%. [66,67]

While BMI is correlated with body fat, it is not a measure of body fat or body composition and is useful for general weight classification only. BMI fails to distinguish between individuals in whom the excess weight is fat and those who are particularly muscular. [68] Self-reported BMI is not valid for children under 12 years or adults over 60 years of age as these groups are likely to either not know their height or weight or to report these in error. [69-71] In general, BMI is more valuable when used in conjunction with waist-to-hip circumference ratio or waist circumference measurements, [72] but these measurements were not collected in the four surveys used in this analysis.

### Data and Knowledge Gaps

• There is currently no surveillance system in Canada to monitor the level of physical activity and body composition of children.

• There is currently no collection of measured body composition or physical activity levels of Canadians.

• Physical activities performed at work (e.g. work-related labour activity), at school (e.g. physical education classes) or in the home (e.g. household chores) are not captured through current Canadian surveillance activities.

• There is a gap in the knowledge surrounding the socio-cultural and ecological determinants of physical activity for girls and women of various cultural backgrounds throughout the lifespan.

• Current knowledge on the associations between physical activity, obesity and chronic disease has been derived from studies performed on predominantly Caucasian males. More research is needed to understand these associations among women and minority populations.

• Data and knowledge surrounding the indirect health care costs associated with physical inactivity and obesity are lacking.

### Recommendations

#### Policy Recommendations

• The recognition by governments of all levels that targeted funding for physical activity and physical education within the school system is essential, given the evidence showing that school-based interventions lead to increased participation in physical activity in school-aged children.

• Development and adoption of Canadian standards for measuring, reporting and monitoring body composition in children under 12 years and adults over 60 years of age, since self-reported BMI is not valid in these age groups.

• Development of Canadian standards for measuring and reporting physical activity levels that are valid for the general population, including children and all minority subgroups.

• Multi-sectoral policy interventions (e.g. health, education, urban development, recreation, industry, transportation) that act to decrease the broad systemic barriers to physical activity and healthy weights among women.

• Integrated approaches using behaviour change as a model for lifestyle changes while addressing the issues related to supportive environments for women in various life stages.

• Targeted interventions that aim to decrease the unique barriers of marginalized Canadians (e.g. women, lower income groups, Aboriginal Canadians, older adults and other special populations).

• Recognition of the importance of the psychological determinants of physical inactivity and overweight/obesity and strategies to help women overcome them.

## Notes

The views expressed in this report do not necessarily represent the views of the Canadian Population Health Initiative, the Canadian Institute for Health Information or Health Canada.
